# A systematic review of the literature on telepsychiatry for bipolar disorder

**DOI:** 10.1002/brb3.2743

**Published:** 2022-09-14

**Authors:** Abigail Farrell, Nevita George, Selen Amado, Janet Wozniak

**Affiliations:** ^1^ Clinical and Research Program in Pediatric Psychopharmacology and Adult ADHD Massachusetts General Hospital Boston Massachusetts USA; ^2^ Department of Psychology University of Houston Houston Texas USA; ^3^ Dauten Family Center for Bipolar Treatment Innovation Massachusetts General Hospital Boston Massachusetts USA; ^4^ Clinical Psychology Department University of Massachusetts Boston Boston Massachusetts USA; ^5^ Department of Psychiatry Harvard Medical School Boston Massachusetts USA

**Keywords:** bipolar disorder, telemedicine, telepsychiatry

## Abstract

**Objective:**

Bipolar (BP) disorder is a highly morbid disorder that is often misdiagnosed or undiagnosed and affects a large number of adults and children. Due to the coronavirus disease 2019 public health emergency stay at home orders, most outpatient mental health care was provided via telepsychiatry, and the many benefits of virtual care ensure that this will continue as an ongoing practice. The main aim of this review was to investigate what is currently known about the use of telepsychiatry services in the diagnosis and treatment of BP disorder across the lifespan.

**Method:**

A systematic literature review assessing the use of telepsychiatry in BP disorder was conducted in PubMed, PsychINFO, and Medline.

**Results:**

Six articles were included in the final review. All included articles assessed populations aged 17 years or older. The literature indicates that BP disorder was addressed in telepsychiatry services at a similar rate as in‐person services, reliable diagnoses can be made using remote interviews, satisfaction rates are comparable to in‐person services, telepsychiatry services are able to reach and impact patients with BP disorder, are sustainable, and patient outcomes can improve using a telepsychiatry intervention.

**Conclusions:**

Given the morbidity of BP disorder, the research addressing the telepsychiatry diagnosis and treatment of BP disorder is sparse, with only emerging evidence of its reliability, effectiveness, and acceptance. There is no research assessing the safety and efficacy of telepsychiatry in pediatric populations with BP disorder. Given the morbidity associated with BP disorder at any age, further research is needed to determine how to safely and effectively incorporate telepsychiatry into clinical care for BP adult and pediatric patients.

## Graphical Abstract

A photo has been provided for the graphical understanding. It is the author's understanding that a short summary of the main findings is to accompany this for the table of contents. Here is a brief summary to be added with the graphical abstract: Given the morbidity of BP disorder, the research addressing the telepsychiatry diagnosis and treatment of BP disorder is sparse, with only emerging evidence of its reliability, effectiveness, and acceptance. There is no research assessing the safety and efficacy of telepsychiatry in pediatric populations with BP disorder. Giver the morbidity associated with BP disorder at any age, further research is needed to determine how to safely and effectively incorporate telepsychiatry into clinical care for BP adult and pediatric patients.

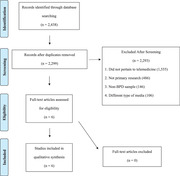

## 1 INTRODUCTION

The outbreak of severe acute respiratory syndrome coronavirus 2, a pathogen that causes coronavirus disease 2019 (COVID‐19), led to a transformation of mental health care. In response to social distancing directives in March 2020, psychiatrists and psychologists as well as other mental health clinicians converted their work from predominantly in‐person encounters to encounters via remote audiovisual platforms, a practice referred to as telepsychiatry. While this change has affected the delivery of health care, these virtual visits are especially well suited to psychiatry, which does not require a hands‐on physical exam.

Telepsychiatry dates back to the 1950s (Chakrabarti, 2015) and the term itself was coined in 1973 (Dwyer, 1973), but its use by state agencies has rapidly increased in recent years, from 15.2% in 2010 to 29.2% in 2017 (Spivak et al., 2020). In 2012, telepsychiatry was reported to be the second most practiced form of virtual health care (Chipps et al., 2012). Our large academic medical center has been utilizing telepsychiatry virtual visits since 2013, with increasing adoption by psychiatrists and psychologists each year and nearly 100% adoption during the early months of the public health emergency March 2020.

Several studies have demonstrated patient and clinician satisfaction with this form of treatment (Hilty et al., 2013; Kroenke et al., 2014; Salmoiraghi & Hussain, 2015) as well as efficacy in a variety of specific patient populations such as underserved minorities, veterans, rural populations, and pediatrics (Lauckner & Whitten, 2016; Spivak et al., 2020; Yeung et al., 2016). While the use of telepsychiatry has been evaluated in several specific disorders including attention‐deficit/hyperactivity disorder (ADHD), anxiety disorders, schizophrenia, and posttraumatic stress disorder (Kasckow et al., 2014; Romijn et al., 2019; Spencer et al., 2020; Sunjaya et al., 2020), it remains unclear whether telepsychiatry can be used safely and effectively in the context of bipolar (BP) disorder.

BP disorder is a highly morbid condition and affects a significant number of adults with an estimated prevalence of 1.3%−5.0% (Dome et al., 2019) in addition to its recognized prevalence in youth of 2.9%–3.9% (Biederman et al., 2001; Lewinsohn et al., 1995; Merikangas et al., 2010; Van Meter et al., 2019). BP disorder affects up to 4.3% of primary care patients (Cerimele et al., 2014) and comprises a significant portion of mental health clinic populations. Because of its morbidity, these patients require intensive psychiatry and psychology services. Diagnosing BP disorder can be difficult (Berk et al., 2005; Smith & Ghaemi, 2010) and only 52% of adults with BP disorder are accurately diagnosed by their first or second mental health professional (Lish et al., 1994). A significant minority of adults with BP disorder, 34%, are not accurately diagnosed for 10 years of illness or longer (Lish et al., 1994). This may be because clinicians often diagnose and treat depression without recognizing mania (Smith et al., 2010). In fact, 25% of adults with BP disorder are initially given a diagnosis of unipolar depression (Lish et al., 1994), and it is estimated that up to 30% of patients presenting with depression are better diagnosed as having BP disorder (Piver et al., 2002). The problem of underdiagnosis in youth is even worse. Pediatric onset BP disorder was largely neglected until recent decades. Children with BP disorder present with features different from the classic presentation (Geller & Luby, 1997; Singh, 2008), and many clinicians still doubt whether BP disorder can present in prepubertal or early adolescent youth (Geller et al., 2004; Ghaemi & Martin, 2007).

Telepsychiatry reduces barriers to psychiatric treatment, making contact with mental health professionals convenient, with briefer interruptions to daily life and work routines. Traveling to appointments with children is especially stressful, and telepsychiatry improves access to mental health care for pediatric populations. Telepsychiatry is especially well suited to the treatment of BP disorder, as these patients often need frequent mental health visits for titration of multiple medications to treat mania, depression, and the many comorbid conditions. In addition, frequent visits for safety monitoring of the pharmacotherapy for BP disorder is necessary due to high rates of adverse events seen in mood stabilizing medications (Baldessarini et al., 2019; Liu et al., 2011). BP disorder carries high rates of suicide, 4%–19% (Dome et al., 2019), requiring regular risk assessment visits. Although telepsychiatry is uniquely well suited to assist in the treatment of BP disorder in adults and children, questions remain as to whether these visits can be conducted safely and effectively via telemedicine.

To this end, we conducted a systematic literature review in order to investigate what is currently known about the use of telepsychiatry services in the treatment of BP disorder across the lifespan.

## 2 METHODS

### 2.1 Database search

We conducted a systematic literature search using PubMed, PsycINFO, and Medline with the search algorithm ((telepsychiatry OR telemedicine OR telehealth) AND (BPD OR bipolar spectrum disorder OR bipolar disorder OR bipolar OR mania)) for articles published through June 23, 2022. References from relevant articles were also reviewed.

### 2.2 Selection criteria

For this literature search, we included articles that investigated the use of telepsychiatry services in relation to BP disorder and that were published in peer‐reviewed journals and written in the English language. Telepsychiatry services were defined as synchronous meetings between a patient and provider for which both audio and visual technology were utilized. Studies involving services delivered asynchronously (e.g., via a mobile phone application or clinician review of patient‐completed rating scales) or using only audio (e.g., check‐ins via phone call) were not included in this review, as these types of services offer different levels and types of care from face‐to‐face telepsychiatry.

We excluded any papers not related to telepsychiatry services, papers that examined a non‐BP disorder sample, papers related to a different type of media (i.e., mobile phone applications or wearable technology), review articles, case reports, and opinion papers. Studies examining a non‐BP disorder sample were defined as those studies which either had a primary focus other than BP disorder or studies which did not explicitly report on a subset of individuals with a diagnosis of BP disorder. The senior author and the lead author screened the articles by abstract for relevance and eligibility. We then reviewed the full texts of articles appearing to be eligible based on the abstract review for inclusion.

## 3 RESULTS

**FIGURE 1:**
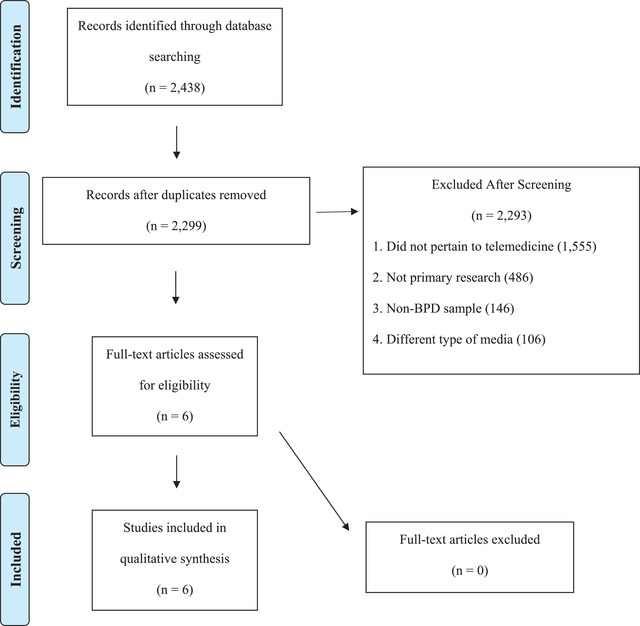
PRISMA diagram: Summary of databases used and articles deemed ineligible with explanations

As shown in Figure 1, our search identified 2438 articles (100 PubMed articles, 1869 PsycINFO articles, 30 MEDLINE articles, and 439 cross‐referenced articles), 139 of which were duplicates. From the 2299 potential articles remaining after duplicates were removed, only six met our inclusion and exclusion criteria following abstract review. We excluded 1555 articles because they did not relate to telepsychiatry services, 146 articles because they examined a non‐BP disorder sample, 106 articles because they were related to a different type of media, and 486 articles because they were theoretical papers.

Of the studies excluded from this analysis, and those that examined a non‐BP disorder sample, a large number of these studies examined a sample with schizophrenia (N = 41) or symptoms of psychosis (N = 13). Four studies included samples with multiple diagnoses and five included those with serious/severe mental illness. None of these studies were included because they either did not specifically include any participants diagnosed with BP disorder (N = 6) or were primarily focused on an aspect of health other than BP disorder (N = 3; i.e., smoking cessation, weight loss). There was a small number of studies (N = 4) which included participants with both BP disorder and posttraumatic stress disorder (PTSD). These were not included because they did not report results specific to the participants with BP disorder. Another 13 studies involved samples with depression, either alone or with participants with one other type of psychiatric diagnosis (i.e., anxiety, psychosis, schizophrenia). These were not included as BP disorder involves additional morbidity and risks associated with manic symptomology. Additionally, 22 examined a non‐psychiatric sample (i.e., surgical or otherwise medical), one focused on providers rather than patients, 13 examined general mental health (i.e., no disorders specified), 24 examined other non‐related psychiatric disorders (i.e., anxiety, autism spectrum disorder, ADHD, PTSD, eating disorders, borderline personality disorder), and five were specifically related to suicide and one to self‐harm behavior.

Of the excluded studies that examined a different type of media, four evaluated various types of wearable technology (i.e., actigraphy), 15 involved web‐based information (i.e., psychoeducation, medical information), another five involved use of social media or open forums, and four dealt with general access to technology. An additional 26 studies dealt with smartphone or other technology‐based monitoring, whether monitoring behaviors, activity, or mood symptoms, rather than diagnostic or interventional approaches. Two studies also assessed monitoring along with wearable technology. There were several intervention‐based studies in this group; seven involved interventions (i.e., ecological momentary intervention) or intervention‐adjacent services (i.e., reminders) using phone calls or text messages and 43 assessed smartphone applications or other interventions delivered via technology or a certain device.

After reviewing the full texts of the six remaining articles, no article were excluded. Therefore, we included six articles in our final literature review.

The characteristics of each study included in this review are presented in Table 1. Almost all of the identified studies included only adult patients; only one study included some pediatric patients, and the youngest participant was 17 years old (Sankar et al., 2021). Three studies provided information on the age of the patients receiving mental health care. Based on these studies, the age range of patients was 17 (Sankar et al., 2021) to 83 years old (Bauer et al., 2016). Three of the studies provided information on the gender of the patients. These samples were 64.38% (Seidel & Kilgus, 2014), 81.41% (Bauer et al., 2016), and 10% (Sankar et al., 2021) male, respectively.

**TABLE 1:** Summary of studies included in systematic literature review on the use of telemedicine services in relation to bipolar (BP) disorders

Paper	Sample	Telepsychiatry description	Measurements	Main findings
Grubbs et al. (2015)	All 11,906,114 mental health encounters (11,729,868 face‐to‐face encounters and 176,246 interactive video encounters) with a diagnosis code in the Veterans Healthcare Administration from October 1, 2011 to September 30, 2012	Mental health encounters delivered via interactive video	Percentage of face‐to‐face encounters versus interactive video encounters by diagnosis	1.7% of all BP disorder encounters were completed via interactive video 5.2% of all face‐to‐face encounters and 6.2% of all interactive video encounters were attributed to diagnoses of BP disorder Interactive video encounters were more likely to address PTSD, depressive disorders, or anxiety disorders
Ruskin et al. (1998)	30 psychiatric inpatients	Structured Clinical Interview for DSM‐III‐R completed via audiovisual telecommunication	Interrater reliability and patient satisfaction scale	BP disorder diagnosis was equally as reliable when conducted in person (interrater reliability = 0.76) and remotely (interrater reliability = 0.81) There were no differences in patient satisfaction between in‐person and remote interviews
Seidel and Kilgus (2014)	73 patients aged over 18 years who presented voluntarily in the emergency department	30‐min assessment through videoconferencing with psychiatrist	Agreement between raters on: DSM‐IV Axis I diagnosis, Beck Scale for Suicide Ideation, HCR‐20 Dangerousness Scale, and recommendation to discharge or hospitalize	No significant differences in the agreement of the two interviewers’ diagnoses and assessments of suicidal ideation, dangerousness, or need for hospitalization between the face‐to‐face condition and the telemedicine condition
Bauer et al. (2016)	The first 400 patients with BP disorder in the VA system who completed intakes for the Bipolar Disorders Telehealth Program	The Bipolar Disorders Telehealth Program (complements local care through clinical video teleconferencing, including a structured diagnostic assessment, pharmacological consultation, seven weekly life goals self‐management sessions, and follow‐up monitoring)	Participation rates and effects on manic, depressive, and conflict symptoms and mental quality of life	Participation rates for the telepsychiatry program were similar to those of the face‐to‐face program on which it was based For program completers, there were significant improvements in manic, depressive, and conflict symptoms and mental quality of life
Bauer et al. (2018)	16 providers involved in the Bipolar Disorders Telehealth Program and the sites at which it has been implemented	The Bipolar Disorders Telehealth Program (complements local care through clinical video teleconferencing, including a structured diagnostic assessment, pharmacological consultation, seven weekly life goals self‐management sessions, and follow‐up monitoring)	Quantitative: implementation and maintenance/ sustainability Qualitative: provider interviews on acceptability	The program grew linearly in the number of participating sites and individuals being served Of the 14 sites that had been participating in the program for at least 2 years, nine sites saw an increase in the use of the program Providers indicated acceptance of the telepsychiatry program Barriers included scheduling difficulties, availability of the equipment and staff needed, and occasional disagreement between consulting providers and primary providers
SANKAR et al. (2021)	13 adolescents/young adults (ages 17–24 years) with bipolar I or II disorder	12‐week social rhythm therapy delivered primarily via telehealth 3 sessions took place in person and nine sessions via video teleconference	Primary outcomes: retention rate, client satisfaction scores, therapeutic alliance ratings Secondary outcomes: social rhythm irregularities, mood symptoms, and suicide propensity	Social rhythm therapy delivered primarily via telehealth had a high retention rate, high client satisfaction scores, and high ratings of therapeutic alliance There were improvements in social rhythm irregularities, mood symptoms, and suicide propensity

Abbreviations: PTSD, posttraumatic stress disorder; VA, Veterans Affairs.

All studies used video conferencing methods to provide telepsychiatry services. Of the five studies included in this review, three looked at use of telepsychiatry across multiple mental health disorders including BP disorder (Grubbs et al., 2015; Ruskin et al., 1998; Seidel & Kilgus, 2014) and three looked at only patients with BP disorder (Bauer et al., 2016, 2018; Sankar et al., 2021). Two of the studies examining only BP disorder patients were part of the Bipolar Disorders Telehealth Program established by the U.S. Department of Veterans Affairs (VA) to expand outpatient care to veterans (Bauer et al., 2016, 2018). The other was part of a larger research study entitled “Brain Emotion Circuitry‐Targeted Self‐Monitoring and Regulation Therapy (BE‐SMART),” which was designed for adolescents and young adults with BP disorder and also included imaging procedures (Sankar et al., 2021). Of the other studies, one was conducted with data on relevant mental health encounters in an outpatient program in the Veterans Health Administration (VHA) (Grubbs et al., 2015), one was conducted with psychiatric inpatients (Ruskin et al., 1998) and one was conducted with psychiatric patients presenting in the emergency department (Seidel & Kilgus, 2014).

The efficacy and acceptability of telepsychiatry services were assessed using a variety of different methods across the different studies. For example, telepsychiatry services were evaluated using quantitative (implementation and maintenance/sustainability) and qualitative (provider interview data) assessments (Bauer et al., 2018), retention rates and quantitative scales related to client satisfaction and therapeutic alliance as well as improvements in mood and social rhythms (Sankar et al., 2021), records of telepsychiatry participation and clinical impact (Bauer et al., 2016), and comparative participation in face‐to‐face versus telepsychiatry settings (Grubbs et al., 2015). These studies also differed in their measurements of interest, as two studies took suicide propensity or suicidal ideation and risk into consideration (Sankar et al., 2021; Seidel & Kilgus, 2014), while another also assessed patient satisfaction at the end of the intervention (Ruskin et al., 1998). Through these various assessment methods, these five studies were able to create a multi‐faceted, although limited, review of the benefits of telepsychiatry services in providing psychiatric care to adult patients with BP disorder.

In one study, Grubbs et al. (2015) investigated which psychiatric diagnoses are most commonly treated using telepsychiatry services. To do so, they compared the diagnoses associated with face‐to‐face encounters with the diagnoses associated with interactive video encounters in the VHA. The authors compiled all 11,906,114 mental health encounters (11,729,868 face‐to‐face encounters and 176,246 interactive video encounters) with a diagnosis code from October 1, 2011 to September 30, 2012 and classified them according to diagnosis. Note that 1.7% of all BP disorder encounters were completed via interactive video. Further, 5.2% of all face‐to‐face encounters and 6.2% of all interactive video encounters were attributed to diagnoses of BP disorder. While interactive video encounters were more likely to address PTSD, depressive disorders, or anxiety disorders, the proportion addressing BP disorder was similar to the proportion of face‐to‐face encounters addressing the same sample, providing indirect evidence of the acceptance of telepsychiatry by clinicians and patients in the treatment of BP disorder.

In another study, Ruskin et al. (1998) examined the reliability of psychiatric diagnoses of BP disorder, major depression, panic disorder, and alcohol dependence made via remote interviews compared to those made via in‐person interviews. Thirty patients in a psychiatric inpatient unit were each interviewed twice just 1 or 2 days apart by the same two trained interviewers using the Structured Clinical Interview for DSM‐III‐R. Half of the patients completed both interviews in person, while the other half completed one in person and one via audiovisual telecommunication. The interrater reliabilities for diagnoses of BP disorder made via two in‐person interviews and one in‐person interview combined with one remote interview were 0.76 and 0.81, respectively, suggesting that remote interviews are as reliable as in‐person interviews in making diagnoses of BP disorder in a population of adult psychiatric inpatients. Furthermore, the patients who completed both in‐person and remote interviews indicated no differences in satisfaction between the two interview forms.

Seidel and Kilgus (2014) later used a similar method to compare the agreement between psychiatric diagnoses for 73 patients over the age of 18 presenting to the emergency department made via face‐to‐face assessment and via video telemedicine assessment. All eligible patients were assessed by one psychiatrist, either face‐to‐face or using videoconferencing equipment, while a second psychiatrist observed face‐to‐face and made an independent assessment. Based on their 30‐min assessment, each psychiatrist reported on a primary DSM‐IV Axis I diagnosis (including BP disorder, depressive disorder, anxiety disorder, psychotic disorder, substance use disorder, or other disorder) as well as suicidal ideation, dangerousness, and their recommendation to discharge or hospitalize. There were no significant differences in the agreement of the two interviewers’ diagnoses and assessments of suicidal ideation, dangerousness, or need for hospitalization between the face‐to‐face condition and the telemedicine condition. These authors concluded that remote video telemedicine assessments can be reliably and safely employed in the emergency department setting.

In a study of the Bipolar Disorders Telehealth Program, Bauer et al. (2016) evaluated both the feasibility and clinical impact of a clinical video teleconferencing program for BP disorder care within the VA system. In this program, clinical procedures included structured diagnostic assessment, psychopharmacologic consultation, seven weekly life goals self‐management sessions, and follow‐up monitoring. In the first 400 patients who completed intakes for the program, participation rates for the telepsychiatry program were similar to those of the face‐to‐face program on which it was based. For those who completed the telepsychiatry program, there were significant improvements in manic, depressive, and conflict symptoms and mental quality of life. Additionally, the estimated suicide attempt rate per year was 2.2% during enrollment in the telepsychiatry program, similar to the estimated rate of 2% stemming from summary data in BP disorder samples (Baldessarini et al., 2001). These results suggest that telepsychiatry‐based care modalities can both effectively reach and clinically impact those with BP disorder. Bauer et al. (2018) later completed a follow‐up analysis of this program, evaluating the extent of its implementation, sustainability, and acceptability among providers. After almost 5 years, the program grew linearly in both the number of participating sites and individuals being served, indicating increased implementation and reach over time. Of the 14 sites that had been participating in the program for at least 2 years, nine sites saw an increase in the use of the program, demonstrating a long‐term sustainability of the program once it has been implemented. Additionally, providers indicated acceptance of the telepsychiatry program, although there were barriers including scheduling difficulties, availability of the equipment and staff needed, and occasional disagreement between consulting providers and primary providers.

Finally, in the most recent study, Sankar et al. (2021) evaluated the feasibility and acceptability as well as the efficacy of a social rhythm therapy (SRT) delivered primarily via telehealth to adolescents and young adults with BP disorder. For this 12‐week manualized treatment, three sessions (first, middle, and last) were conducted in person and the remaining nine sessions were conducted via a video teleconferencing platform. This SRT treatment had a retention rate of 77%. Client satisfaction was measured using the client satisfaction questionnaire, and the mean score of 29.4 was noted to be excellent. Additionally, therapeutic alliance quality was measured by the Working Alliance Inventory, and mean scores on all subscales (task, bond, and goal) from both the therapists and participants were high. Secondary outcomes also included clinical measures, and improvements were found across the board in terms of mood symptoms, social rhythm irregularities, and suicide propensity from pre‐ to post‐intervention time points.

## 4 DISCUSSION

Our systematic literature review on the current knowledge about the use of telepsychiatry services in relation to BP disorder identified six articles. We excluded 252 articles that did not address BP disorder specifically (146 articles) or focused on alternative types of media beyond synchronous clinician–patient virtual visits (106 articles), as detailed above, which demonstrates the heterogeneous approach to studying telepsychiatry across diverse modalities, varied diagnostic populations, and with a variety of safety and outcome measures. This state of affairs highlights the urgent need for additional systematic research addressing the safety and efficacy of this increasingly popular modality of diagnosis and treatment, especially in populations with severe psychiatric disorders such as bipolar disorder. Knowledge gained from these differing studies is important in evaluating accessible and convenient methods of providing clinical care to large numbers of patients. These studies, however, were not included in the present review as we aimed to evaluate the safety and efficacy of synchronous clinician‐based, audio‐visual services (what are commonly called “virtual visits”), which expanded in use during the public health emergency and, due to their convenience and popularity, will continue to be implemented in treatment plans. In addition, we chose studies specifically addressing BP disorder as this population carries especially high risks of suicide, substance use disorders, and conduct/antisocial behaviors which pose large personal and public health risks.

It is expected that the public health emergency caused by COVID‐19 will result in persistent changes to treatment delivery, including an increase in the use of telepsychiatry. In fact, de Siqueira Rotenberg et al. (2020) have published a recommendation for a “swift transition” to digitally based approaches for the treatment of BP disorder. Therefore, it is critical to understand what is gained and what is lost during the delivery of treatment using telepsychiatry in place of traditional in‐person visits. The very sparse existing literature shows that telepsychiatry may be utilized as frequently as in‐person services for adults with BP disorder, indicating acceptance by clinicians and patients (Grubbs et al., 2015). Additional evidence supports telepsychiatry as reliable for the purpose of accurate diagnosis of BP disorder in adults, but this was studied only in psychiatric inpatient and ED settings (Ruskin et al., 1998; Seidel & Kilgus, 2014). For psychiatric inpatients with BP disorder, telepsychiatry was found to be as satisfactory as in‐person services (Ruskin et al., 1998), consistent with recent reports of acceptability of other forms of digital health interventions among patients with serious mental health problems, but this satisfaction survey was based on only one virtual visit done in conjunction with an in‐person visit (Berry et al., 2019; Vaidyam et al., 2019). Psychotherapy services delivered via video teleconferencing methods were found to be feasible and acceptable (as measured by retention rates, client satisfaction, and therapeutic alliance ratings) and to be clinically effective for mood, social rhythms, and suicidality in older adolescents and young adults (Sankar et al., 2021). However, this study provided no direct comparison to similar services provided in person. Finally, the most extensive use of telepsychiatry services was in the VA system. In this extensive 6‐month time‐limited treatment program, telepsychiatry was found to be clinically effective and feasible, indicated by increased implementation, sustainability over time, and acceptability among providers (Bauer et al., 2016, 2018). Some of the articles excluded from review for various reasons also provided useful information about the effectiveness of telepsychiatry for more general populations including those with BP disorder (Fortney et al., 2021). Taken together, this emerging evidence supports the idea that telepsychiatry services can be used safely and effectively for the treatment of older adolescents and adults with BP disorder, but the evidence is sparse and leaves many questions unanswered for this highly morbid population.

The role of telepsychiatry in providing timely and accurate BP diagnoses merits further study. Given research that BP disorder may be difficult to diagnosis, with patients seeking care for years prior to receiving an accurate diagnosis, telepsychiatry may provide an opportunity to improve diagnostic accuracy through the increased use of structured interview questions and patient‐completed questionnaires to augment virtual visits. Virtual visits limit patient–clinician interaction and make parts of the mental status exam difficult to accomplish via observation, meaning that a best practice for clinicians performing virtual visits may be to rely increasingly on direct questioning about symptoms. Such a practice could benefit BP patients who may present in depressed states, masking possible manic symptoms. On the other hand, psychiatric diagnoses of mood disorders including depression and BP disorder rely on the mental status exam which may be limited when done via telepsychiatry.

The existing literature which supports the use of telepsychiatry for the treatment of BP disorder is limited to mainly adult populations, despite the fact that telepsychiatry, which eliminates the stress of travel to appointments, is especially well‐suited to the treatment of children with BP disorder. Not one article identified in this systematic literature review focused on a pediatric population, and the only study including a pediatric population included a small sampling of only older adolescents, with the youngest participant being 17 years old (Sankar et al., 2021). Pediatric psychiatry clinical assessments rely on the interaction of the child patient with their surroundings. Pediatric mental health clinicians observe the physical movements of the child, the connection of the child to their parent and the relatedness of the child to the interviewer, all of which could be impacted by virtual visit formats and could affect diagnosis and assessment. In addition, pediatric BP disorder continues to be considered a difficult diagnosis to establish especially in very young children. Overlap with ADHD, and other comorbidities, such as autism spectrum disorder, complicate the diagnosis. Whether these factors affect assessment or treatment via telepsychiatry is unknown. As with adult psychiatry, if the lack of visual clues for the clinician leads to increasing reliance on structured questions, this could benefit children with BP disorder, who may not show symptoms during the visit. Future studies are needed to assess the use of telepsychiatry for the diagnosis, assessment, and treatment of children with BP disorder.

The current available literature on telepsychiatry treatment of BP disorder is further limited in measuring the wide scope experience of how virtual visits differ from in‐person treatment for both patients and clinicians, affecting the treatment alliance. Sankar et al. (2021) provide evidence that the therapeutic alliance is strong in telepsychiatry‐based services, but these authors did not provide a comparison to in‐person treatment, limiting the ability to draw generalized conclusions. Given the rapid, widespread, and extensive adoption of telepsychiatry during the COVID‐19 public health crisis, future psychiatric assessment and treatment will likely continue to utilize this modality. For clinicians and patients, this brings both gains and losses which have been minimally delineated, and awareness of these can inform more responsible treatment planning and better outcomes.

For example, patient satisfaction may be excellent for telepsychiatry services (Sankar et al., 2021) and may not differ overall between services delivered in person and via telepsychiatry (Ruskin et al., 1998); however, the satisfaction reported by patients may be due to different aspects of the visit. Patients may be more satisfied with the lack of commute and convenience of telepsychiatry but may be less satisfied with their interpersonal connection with the clinician than when in‐person services are employed. Further, while a recent survey indicated that digital health interventions are acceptable among patients with severe mental health problems, there was also evidence of significant fears that digital interventions might replace other mental health services (Berry et al., 2019). Finally, a review of the current literature on digital mental health interventions suggests that, although a therapeutic alliance can be successfully built within this format (Sankar et al., 2021), the characteristics of this alliance and how to best design digital interventions to cultivate a positive alliance remain unclear (Tremain et al., 2020). How these factors might impact adherence to and effectiveness of treatment for BP patients is unknown.

An additional important consideration in high‐risk populations such as BP disorder is safety, including assessment of suicide risk and risk mitigation. Of the six studies reviewed here, three included mention of suicide risk in some way (Bauer et al., 2016; Sankar et al., 2021; Seidel & Kilgus, 2014). In one study comparing a face‐to‐face encounter with a telepsychiatry‐based encounter, assessment of suicidal risk was found to be comparable (Seidel & Kilgus, 2014), providing key initial evidence that risk assessment via telepsychiatry is adequate. Additionally, in the VA's large telepsychiatry program for BP disorder, rates of suicide attempts while enrolled were found to be equal compared to large, aggregated samples of BP disorder patients not in treatment (Bauer et al., 2016). The authors note that it is surprising that suicide attempts were not more frequent in the program due to the increased complexity and unstable nature of the cases that required them to be referred to specialty care. Further, in their evaluation of a telepsychiatry‐based SRT, Sankar et al. (2021) found an improvement in suicide propensity from pre‐ to posttreatment. Overall, there is a significant need to ensure that safety is a priority both when utilizing telepsychiatry and when using traditional face‐to‐face services. Unfortunately, there is little research on this topic, and a recent comprehensive review of suicide risk reported that our ability to predict suicide attempts is only slightly better than chance (Franklin et al., 2017). Further research is certainly needed to improve our ability to maintain patient safety through suicide risk assessment, both in general and specifically in regard to telepsychiatry‐based services, as these continue to increase in popularity.

Whether for children with BP disorder or other populations, deciphering the details of what is better done via telepsychiatry versus in‐person services will allow clinicians to make informed decisions regarding when telepsychiatry is best indicated, for which patients, and during which phase of illness. For this purpose, measures of interest might include parsing the content of what occurs during each type of appointment, how quickly treatment plans are created and implemented, and how much value each visit adds to the patient's recovery. While the Bipolar Disorders Telehealth Program and SRT studies demonstrated the clinical efficacy of telepsychiatry (Bauer et al., 2016; Sankar et al., 2021), there was no indication of how this compared to clinical improvements seen when in‐person services are employed, or when a combination of in‐person and telepsychiatry visits are used. Research which directly compares the clinical efficacy of telepsychiatry visits versus in‐person services can improve trust in telepsychiatry by demonstrating that it is on par with traditional practice. In fact, a recent review of telepsychiatry directly compared to in‐person services similarly found no existing studies focused on a BP disorder population, and these authors also noted the need for such upcoming research (Greenwood et al., 2022).

In conclusion, the very small amount of emerging evidence on telepsychiatry indicates that it holds to promise to be a reliable, effective, and accepted method of delivering services for BP disorder in older adolescent and adult populations. However, further research is required to assess whether its demonstrated utility expands to pediatric populations, who could benefit greatly from telepsychiatry services, and to further investigate for each population exactly what is gained and what is lost when delivering services via telepsychiatry versus in person. Such information is key to the successful development of hybrid treatment plans combining virtual and in‐person care.

## CONFLICT OF INTEREST

Dr. Janet Wozniak receives research support from PCORI and Demarest Lloyd, Jr. Foundation. In the past, Dr. Wozniak has received research support, consultation fees or speaker's fees from Eli Lilly, Janssen, Johnson and Johnson, McNeil, Merck/Schering‐Plough, the National Institute of Mental Health (NIMH) of the National Institutes of Health (NIH), Pfizer, and Shire. She is the author of the book, “Is Your Child Bipolar” published May 2008, Bantam Books. Her spouse receives royalties from UpToDate; consultation fees from Emalex, Noctrix, Disc Medicine, Avadel, HALEO, OrbiMed, and CVS; and research support from Merck, NeuroMetrix, American Regent, NIH, NIMH, the RLS Foundation, and the Baszucki Brain Research Fund. In the past, he has received honoraria, royalties, research support, consultation fees or speaker's fees from Otsuka, Cambridge University Press, Advance Medical, Arbor Pharmaceuticals, Axon Labs, Boehringer‐Ingelheim, Cantor Colburn, Covance, Cephalon, Eli Lilly, FlexPharma, GlaxoSmithKline, Impax, Jazz Pharmaceuticals, King, Luitpold, Novartis, Neurogen, Novadel Pharma, Pfizer, Sanofi‐Aventis, Sepracor, Sunovion, Takeda, UCB (Schwarz) Pharma, Wyeth, Xenoport, Zeo. Abigail Farrell, Nevita George, and Selen Amado declare no conflict of interest.

### PEER REVIEW

The peer review history for this article is available at: https://publons.com/publon/10.1002/brb3.2743.

## Data Availability

Data sharing not applicable to this article as no datasets were generated or analyzed during the current study.
